# Histidine Deficiency Inhibits Intestinal Antioxidant Capacity and Induces Intestinal Endoplasmic-Reticulum Stress, Inflammatory Response, Apoptosis, and Necroptosis in Largemouth Bass (*Micropterus salmoides*)

**DOI:** 10.3390/antiox11122399

**Published:** 2022-12-02

**Authors:** Hualiang Liang, Pao Xu, Gangchun Xu, Lin Zhang, Dongyu Huang, Mingchun Ren, Lu Zhang

**Affiliations:** 1 Key Laboratory of Integrated Rice-Fish Farming Ecology, Ministry of Agriculture and Rural Affairs, Freshwater Fisheries Research Center, Chinese Academy of Fishery Sciences, Wuxi 214081, China; 2 Wuxi Fisheries College, Nanjing Agricultural University, Wuxi 214081, China; 3 Healthy Aquaculture Key Laboratory of Sichuan Province, Tongwei Co., Ltd., Chengdu 610093, China

**Keywords:** histidine deficiency, juvenile largemouth bass (*Micropterus salmoides*), antioxidant capacity, endoplasmic-reticulum stress, inflammation

## Abstract

This 56-day study aimed to evaluate the effects of histidine levels on intestinal antioxidant capacity and endoplasmic-reticulum stress (ERS) in largemouth bass (*Micropterus salmoides*). The initial weights of the largemouth bass were (12.33 ± 0.01) g. They were fed six graded levels of histidine: 0.71% (deficient group), 0.89%, 1.08%, 1.26%, 1.48%, and 1.67%. The results showed that histidine deficiency significantly suppressed the intestinal antioxidant enzyme activities, including SOD, CAT, GPx, and intestinal level of GSH, which was supported by significantly higher levels of intestinal MDA. Moreover, histidine deficiency significantly lowered the mRNA level of *nrf2* and upregulated the mRNA level of *keap1*, which further lowered the mRNA levels of the downstream genes *sod*, *cat,* and *gpx*. Additionally, histidine-deficiency-induced intestinal ERS, which was characterized by activating the PEPK-signalling pathway and IRE1-signalling pathway, including increased core gene expression of *pepk*, *grp78*, eif2α, *atf4*, *chopα*, *ire1*, *xbp1*, *traf2*, *ask1,* and *jnk1*. Dietary histidine deficiency also induced apoptosis and necroptosis in the intestine by upregulating the expressions of proapoptotic genes, including *caspase 3*, *caspase 8*, *caspase 9*, and *bax,* and necroptosis-related genes, including *mlkl* and *ripk3*, while also lowering the mRNA level of the antiapoptotic gene *bcl-2*. Furthermore, histidine deficiency activated the NF-κB-signalling pathway to induce an inflammatory response, improving the mRNA levels of the proinflammatory factors *tnf-α*, *hepcidin 1*, *cox2*, *cd80*, and *cd83* and lowering the mRNA levels of the anti-inflammatory factors *tgf-β1* and *ikbα*. Similarly, dietary histidine deficiency significantly lowered the intestinal levels of the anti-inflammatory factors TGF-β and IL-10 and upregulated the intestinal levels of the proinflammatory factor TNF-α, showing a trend similar to the gene expression of inflammatory factors. However, dietary histidine deficiency inhibited only the level of C3, and no significant effects were observed for IgM, IgG, HSP70, or IFN-γ. Based on the MDA and T-SOD results, the appropriate dietary histidine requirements of juvenile largemouth bass were 1.32% of the diet (2.81% dietary protein) and 1.47% of the diet (3.13% dietary protein), respectively, as determined by quadratic regression analysis.

## 1. Introduction

The intestine, as one of the most important tissues in fish, plays a role in the digestion and absorption of nutrients; hence, intestinal health is a prerequisite for healthy animal growth [1,2]. The intestinal tract is also the largest mucosal immune organ and the most vulnerable immune barrier in fish [3]. However, fish intestinal walls are thin and easily damaged [2]. Additionally, the intestinal polyunsaturated fatty acid content in fish is high and easily damaged by oxidation [1,2,3]. In addition, the intestinal tract is one of the parts of the body most closely related to the external environment and is prone to environmental stress. Therefore, it is important to construct nutritional programs to maintain the intestinal health of fish. Numerous studies have shown that healthy intestinal development depends on the composition of the feed formulation, including elements such as amino acids [4,5], tributyrin [6], and vitamins [7,8]. These studies suggested that balanced nutrition may have beneficial effects on the intestinal health of fish.

Histidine, an essential amino acid for fish, has important functions in improving fish growth [9]. According to research reports, histidine acts as an antioxidant and plays an important role in scavenging oxidative free radicals in fish and mammals [10,11]. Histidine has other significant antioxidant activities in addition to scavenging free radicals, such as binding divalent metal ions and resisting glycation [10,12]. In grass carp, dietary histidine has a positive effect on lowering lipid peroxidation and protein oxidation in the intestines and gills [4,13]. In addition, histidine has important functions in inhibiting lipid peroxidation in the sarcoplasmic reticulum of fish muscle [14]. The antioxidant functions of histidine are mainly achieved through the nuclear factor erythroid 2-related factor 2 (Nrf2) signalling pathway [13,15]. Furthermore, the endoplasmic reticulum (ER), as one of the most important organelles in eukaryotic cells, is closely related to cell homeostasis, and ER homeostasis is maintained through unfolded protein reactions involving three signalling pathways: the PEPK-signalling pathway, IRE-1-signalling pathway, and ATF6-signalling pathway [16]. In aquatic animals, numerous studies have reported that adverse factors could induce endoplasmic-reticulum stress (ERS) by affecting the mRNA levels of core genes in the IRE1-signalling pathway, PERK-signalling pathway, and ATF6-signalling pathway, such as high-fat induction in blunt snout bream (*Megalobrama amblycephala*) [17], hydrogen peroxide exposure and ammonia stress in common carp (*Cyprinus carpio*) [18,19], waterborne cadmium exposure in Gibel carp (*Carassius gibelio*) [20], and nitrite induction in grass carp (*Ctenopharyngodon idella*) [21]. When ERS occurs or is persistent and exceeds the range of cellular self-regulation, it leads to cell apoptosis and death and induces disease [22]. Furthermore, ERS might activate the NF-κB-signalling pathway and regulate downstream inflammatory factors to induce an inflammatory response [23]. Hence, reducing ERS and oxidative stress is crucial to improving animal health and immunity.

The largemouth bass (*Micropterus salmoides*) is native to freshwater rivers and large lakes in America, especially the Great Lakes in the United States. These fish have been widely raised around the world because of their tender meat and delicious taste that is loved by consumers. Largemouth bass sell well on the international market and are known as freshwater grouper. However, due to the problems of breeding density and nutrient level, largemouth bass often suffer from physiological problems such as metabolic disorders and intestinal inflammation, which could reduce their growth and induce diseases, resulting in great economic losses. In our previous study, the appropriate dietary histidine level was 1.26% of their diet [24], which showed the best growth performance. However, how histidine, an important antioxidant, regulates the intestinal health needs to be investigated. Hence, this study is to assess the functions of histidine in the intestinal health of largemouth bass.

## 2. Materials and Methods

### 2.1. Ethical Statement

This experiment was based strictly on the requirements of the Institutional Animal Care and Ethics Committee of Nanjing Agricultural University, Nanjing, China [Permit number: SYXK (Su) 2011-0036].

### 2.2. Experimental Diet

The experimental formulation referred to our previous study [24]. A combination of crystalline amino acids, excluding histidine, was supplemented to simulate the 47%-whole-body-amino-acid pattern of largemouth bass [24]. The additional level of histidine was designed with an arithmetic difference of 0.2%. The compositional analysis of histidine levels in feed were 0.71%, 0.89%, 1.08%, 1.26%, 1.48%, and 1.67%. The basic formula levels are shown in Table 1. All ingredients were fully crushed to completely pass through an 80 μm mesh screen, and then the feed was prepared according to the experimental formulas and thoroughly mixed with water and oil. Lastly, the mixed ingredients were made into 1 mm expanded pellets with an aquatic feed puffing machine (Jiangsu Muyang Holdings Co., Ltd., Yangzhou, China). The feed was dried and stored for breeding experiments.

### 2.3. Breeding Experiment

Juvenile largemouth bass ((12.33 ± 0.01) g) were randomly divided into 18 experimental cages (3 replicates for each treatment group and 20 fish in each experimental cage). The fish were fed two times daily (at 7:00 and 17:00) with the appropriate DHL feed according to the standard of apparent satiety. The indices of the aquaculture water during the experiment were (28 ± 2) °C (water temperature), 7–7.5 (pH), >6.0 mg/L (dissolved oxygen), and <0.01 mg/L (total ammonia nitrogen).

### 2.4. Sample Collection

At the end of 56 days of feeding, the fish were starved for 24 h. After anesthetization with MS-222 (200 mg/L), three fish from each cage were dissected to obtain intestine samples, and a total of 9 samples in each experimental group were used for antioxidant indices, immune cytokines, and qRT-PCR analysis.. These samples were stored at −80 °C for subsequent analysis.

### 2.5. Chemical Analysis, RNA Extraction, and Quantitative Real-Time PCR

Measurements of the moisture, crude protein, ash, and crude lipid of the raw materials and diets were conducted using the established methods of AOAC [25]. After pretreatment, all samples were analyzed using an Agilent-1100 amino acid assay system (Agilent Technologies Co., Ltd., Santa Clara, CA, USA). Tryptophan hydrolysis was carried out in 5 N NaOH at 110 °C for 20 h, which referred to our previous study [24]. The gross energy of diets were analyzed by combustion using a IKA C6000 oxygen bomb calorimeter (IKA WORKS GUANGZHOU, Guangzhou, China). The contents of glutathione (GSH) and malondialdehyde (MDA), as well as the activities of catalase (CAT), glutathione peroxidase (GPx), total superoxide dismutase (T-SOD), and total antioxidant capacity (T-AOC), were analyzed with commercial kits (Nanjing Jiancheng Institutes, Nanjing, China). Enzyme-linked immunosorbent assays (ELISAs) were used to evaluate the contents of intestinal tumour necrosis factor-α (TNF-α), interleukin 10 (IL-10), transforming growth factor-β (TGF-β), heat stress protein 70 (HSP70), interleukin 1β (IL-1β), component 3 (C3), immunoglobulin M (IgM), interleukin 8 (IL-8), and interferon-γ (IFN-γ) by the double-antibody sandwich method (with a test wavelength of 450 nm) according to our previous study [6].

The gene mRNA levels were analysed by qRT-PCR. The main processes included extracting tissue RNA, checking the quantity and quality of the RNA, and proceeding with qRT–PCR analysis on a 7500 Real Time PCR System (Applied Biosystems, Foster city, CA, USA), which was described in our previous study [26]. The designed primers for the qRT-PCR analysis are shown in Table 2. The internal reference gene is glyceraldehyde-3-phosphate dehydrogenase (*gapdh*), and the mRNA levels were calculated based on the standard curve and quantified using a relative standard curve method.

### 2.6. Statistical Analysis

The data were evaluated by one-way analysis of variance (ANOVA) with Tukey’s multiple comparisons test. The results are presented as the means with the standard deviation. Results with *p*-values less than 0.05 indicate that the results are significantly different.

## 3. Results

### 3.1. Intestinal Antioxidant Status

**Figure 1:**
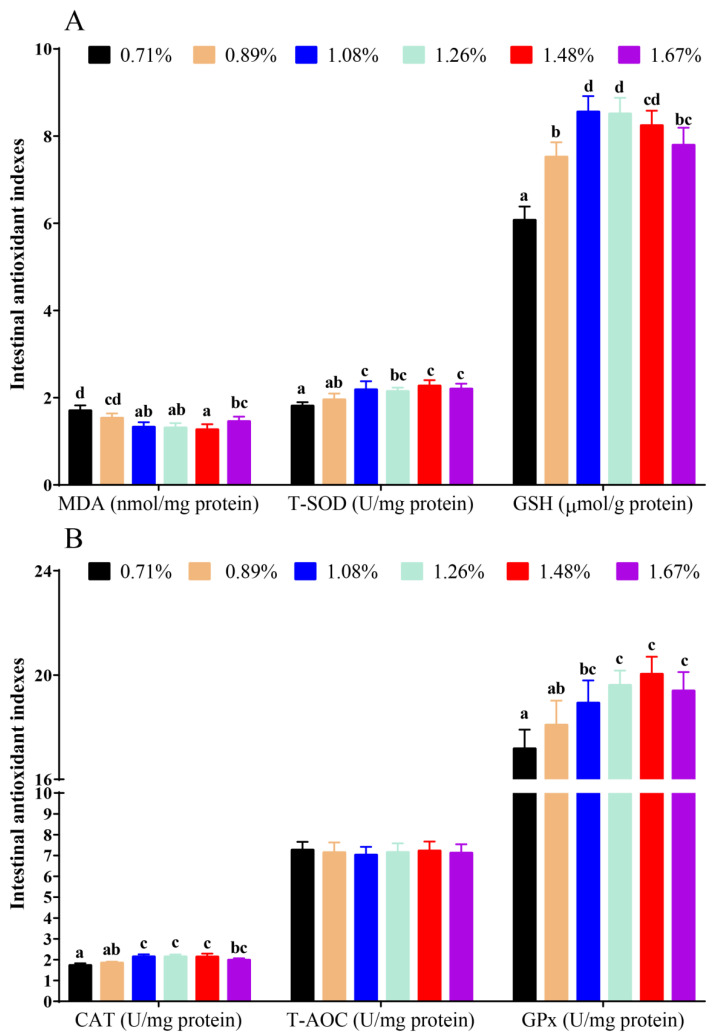
The results of intestinal MAD and antioxidant indexes against graded different levels of dietary histidine. (**A**) showed the results of MDA, T-SOD and GSH, (**B**) showed the results of CAT, T-AOC and GPx. Data are presented as mean ± standard deviation (*n* = 3 × 3). Values with different alphabetical superscripts above bars are significantly different (*p* < 0.05).

**Figure 2:**
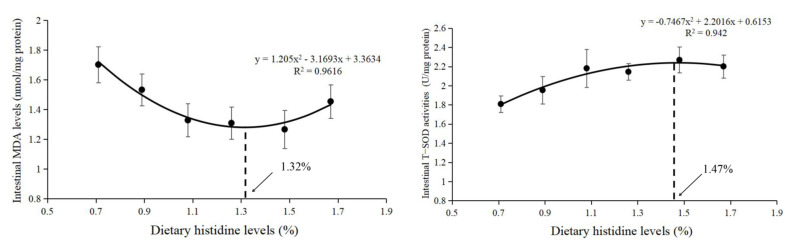
Quadratic regression analysis of MDA and T-SOD against graded different levels of dietary histidine.

Compared with the 0.71% dietary histidine level (DHL), the (1.08–1.67)% DHLs significantly lowered the intestinal MDA levels and upregulated the intestinal antioxidant enzyme activities of GPx, T-SOD, and CAT, and the (0.89–1.67)% DHLs significantly upregulated the intestinal GSH levels (*p* < 0.05) (Figure 1A,B). However, no significant changes in T-AOC were found with different DHLs (*p* > 0.05) (Figure 1B). Based on the MDA and T-SOD results, the dietary histidine requirements of juvenile largemouth bass were 1.32% of the diet (2.81% dietary protein) and 1.47% of the diet (3.13% dietary protein), as determined by quadratic regression analysis (Figure 2).

### 3.2. Intestinal Immune Cytokines

**Figure 3:**
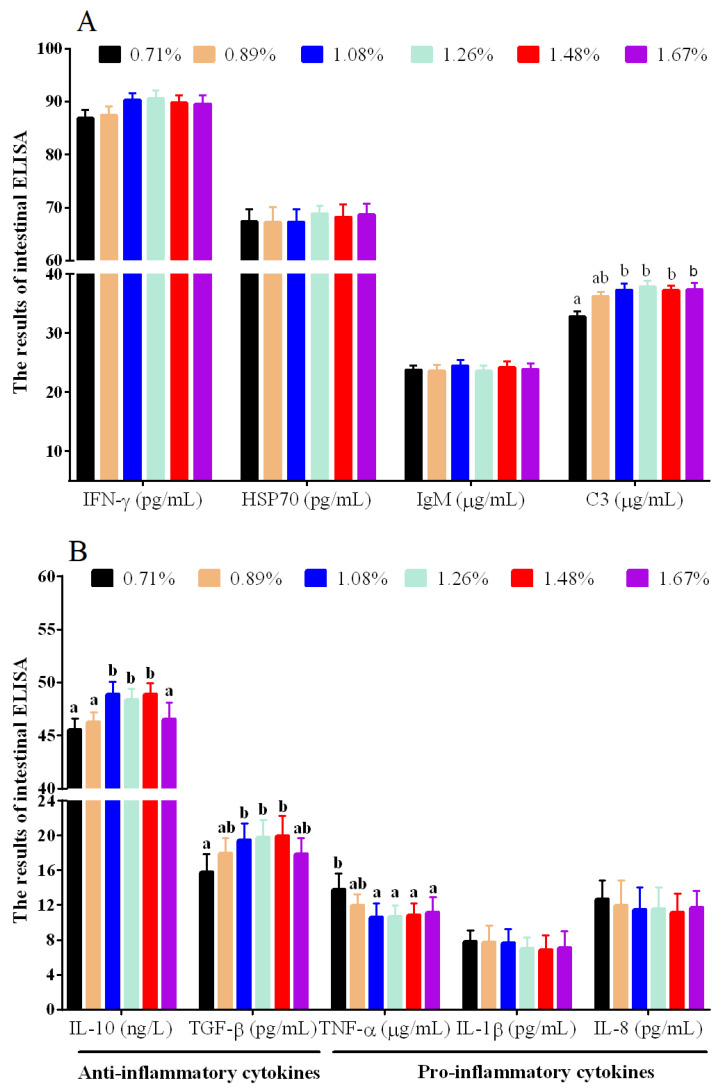
The results of immune cytokines of intestinal ELISA against graded different levels of dietary histidine. (**A**) showed the results of IFN-γ, HSP70, IgM and C3. (**B**) showed the results of IL-10, TGF-β, TNF-α, IL-1β and IL-8. Data are presented as mean ± standard deviation (*n* = 3 × 3). Values with different alphabetical superscripts above bars are significantly different (*p* < 0.05).

Compared with the 0.71% DHL, significantly higher levels of intestinal C3 were observed at the (1.08–1.67)% DHLs (*p* < 0.05) (Figure 3A). DHLs of (1.08–1.48)% significantly upregulated the intestinal anti-inflammatory cytokine levels of TGF-β and IL-10, and the 1.08%–1.67% DHLs significantly lowered the intestinal proinflammatory cytokine level of TNF-α (*p* < 0.05) (Figure 3B). However, DHL had no significant effect on the intestinal levels of the immune cytokines IL-1β, IFN-γ, IgM, HSP70, and IL-8 (*p* > 0.05) (Figure 3A,B).

### 3.3. The mRNA Levels of the Key Genes in the Nrf2-Signalling Pathway

**Figure 4:**
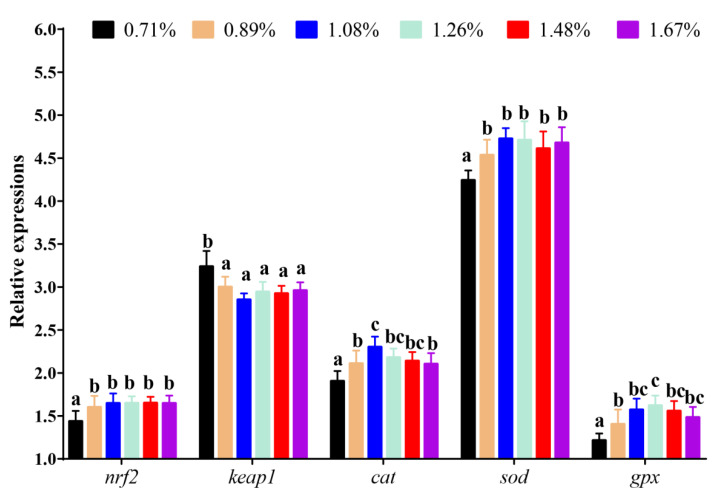
The mRNA levels of the key genes of the Nrf2-signaling pathway against graded different levels of dietary histidine. Data are presented as mean ± standard deviation (*n* = 3 × 3). Values with different alphabetical superscripts above bars are significantly different (*p* < 0.05).

Compared with the 0.71% DHL, significantly higher mRNA levels of *nrf2* were observed in the (0.89–1.67)% DHL groups (*p* < 0.05). However, DHLs of (0.89–1.67)% significantly lowered the mRNA levels of Kelch-like ECH-associated protein 1 (*keap1*) (*p* < 0.05). Furthermore, compared with the control group, the (0.89–1.67)% DHLs also significantly upregulated the mRNA levels of *gpx*, *cat,* and superoxide dismutase (*sod*) (*p* < 0.05) (Figure 4).

### 3.4. The mRNA Levels of Inflammatory-Related Genes

**Figure 5:**
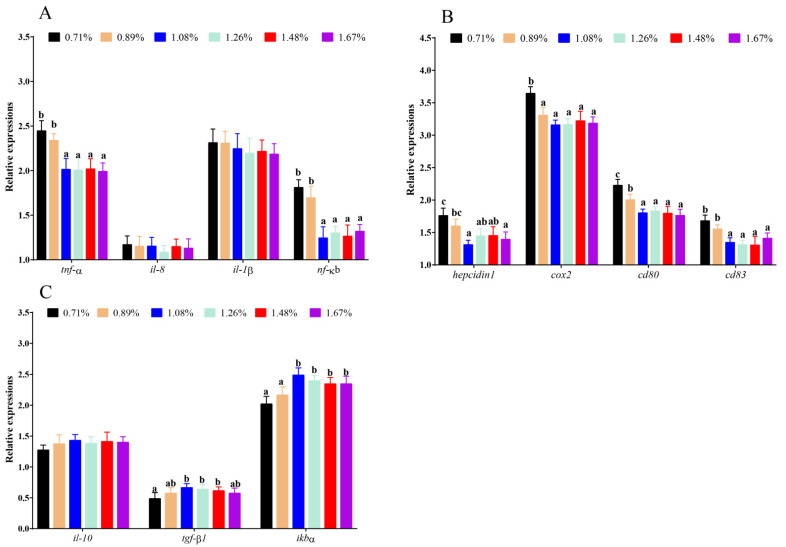
The mRNA levels of inflammatory-related genes against graded different levels of dietary histidine. (**A**) showed the results of *tnf-α*, *il-8*, *il-1β* and *nf-κb*. (**B**) showed the results of *hepcidin 1, cox2, cd80* and *cd83*. (**C**) showed the results of *il-8*, *tgf-β1 and ikbα.* Data are presented as mean ± standard deviation (*n* = 3 × 3). Values with different alphabetical superscripts above bars are significantly different (*p* < 0.05).

Compared with the 0.71% DHL, DHLs of (1.08–1.67)% significantly lowered the mRNA levels of the proinflammatory-related genes nuclear factor kappa B (*nf-κb*), *tnf-α*, *hepcidin 1,* and *cd83* (*p* < 0.05) (Figure 5A,B). However, DHLs of (0.89–1.67)% also lowered the mRNA levels of the proinflammatory-related genes cyclooxygenase 2 (*cox2*) and *cd80* (*p* < 0.05) (Figure 5B). Additionally, the (1.08–1.48)% DHLs significantly upregulated the mRNA levels of the anti-inflammatory-related gene transforming growth factor β1 (*tgf-β1*) (*p* < 0.05), and the (1.08–1.67)% DHLs significantly upregulated the mRNA levels of the anti-inflammatory-related gene NF-kappa-B inhibitor alpha (*ikbα*) (*p* < 0.05) (Figure 5C). However, no significant changes in *il-8*, *il-1β,* or *il-10* were observed in fish fed different DHLs (*p* > 0.05) (Figure 5A,C).

### 3.5. The mRNA Levels of Apoptosis and Necroptosis Genes

**Figure 6:**
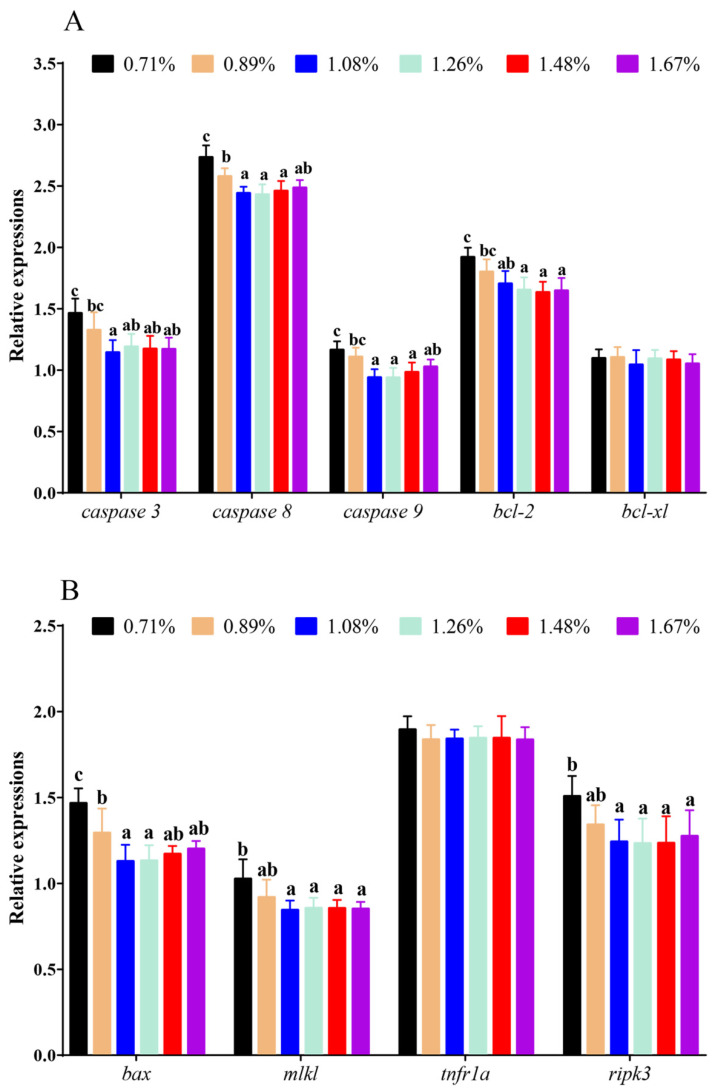
The mRNA levels of apoptosis and necroptosis genes against graded different levels of dietary histidine. (**A**) showed the results of *caspase 3*, *caspase 8*, *caspase 9*, *bcl-2* and *bcl-xl*. (**B**) showed the results of *bax*, *mlkl*, *tnfr1a* and *ripk3.* Data are presented as mean ± standard deviation (*n* = 3 × 3). Values with different alphabetical superscripts above bars are significantly different (*p* < 0.05).

Compared with the 0.71% DHL, significantly lower mRNA levels of B-cell leukaemia/lymphoma 2 (*bcl-2*), *caspase 3*, mixed lineage kinase-like (*mlkl*), *caspase* 9, and receptor-interacting protein kinase-3 (*ripk3*) were observed with the (1.08–1.67)% DHLs (*p* < 0.05), and significantly lower mRNA levels of *caspase 8* and bcl-2-associated x (*bax*) were also observed with the (0.89–1.67)% DHLs (*p* < 0.05) (Figure 6A,B). However, DHL had no significant effect on the mRNA levels of B-cell leukaemia/lymphoma-XL (*bcl-xl*) and tumour necrosis factor receptor 1A (*tnfr1a*) (*p* > 0.05) (Figure 6A,B).

### 3.6. The mRNA Levels of ERS-Related Genes

**Figure 7:**
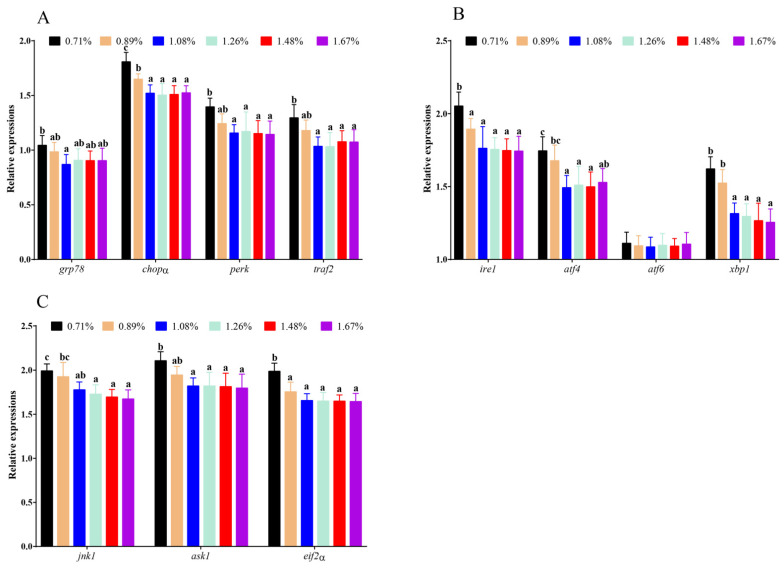
The mRNA levels of endoplasmic-reticulum-stress-related genes against graded different levels of dietary histidine. (**A**) showed the results of *grp78*, *chopα*, *perk* and *traf2*. (**B**) showed the results of *ire1*, *atf4*, *atf6* and *xbp1*. (**C**) showed the results of *jnk1*, *ask1* and *eif2α*. Data are presented as mean ± standard deviation (*n* = 3 × 3). Values with different alphabetical superscripts above bars are significantly different (*p* < 0.05).

Compared with the 0.71% DHL, significantly higher mRNA levels of c-Jun N-terminal kinase-1 (jnk1), activating transcription factor 4 (*atf4*), X-box binding protein 1 (*xbp1*), apoptosis signal-regulating kinase 1 (*ask1*), protein kinase R (PKR)-like endoplasmic-reticulum kinase (*perk*), and TNF receptor-associated factor 2 (*traf2*) were observed with the (1.08–1.67)% DHLs (*p* < 0.05), and significantly higher mRNA levels of eukaryotic translation initiation factor 2 (*eif2α*), C/EBP-homologous protein (*chopα*), and inositol-requiring enzyme 1 (*ire1*) were also observed with the (0.89–1.67)% DHLs (*p* < 0.05) (Figure 7A–C). Furthermore, the 1.08% DHL significantly upregulated the mRNA level of 78 kDa glucose-regulated protein (*grp78*) (*p* < 0.05) (Figure 7A). However, DHL had no significant effect on the mRNA levels of activating transcription factor 6 (*atf6*) (*p* > 0.05) (Figure 7B).

## 4. Discussion

### 4.1. Histidine Deficiency Inhibited Intestinal Antioxidant Capacity

MDA is marker for oxidative stress, which can reflect the degree of cellular oxidative stress [33]. This study found that dietary histidine deficiency (0.71% DHL) significantly upregulated the intestinal MDA level, indicating that dietary histidine deficiency could cause oxidative damage. Similar reports were also found in the serum of Jian carp (*C. carpio* var. Jian) [11], gills of grass carp [13], and muscles of grass carp [15]. The increase in MDA level caused by histidine deficiency may be related to a decreased ability to scavenge superoxide anions and hydroxyl radicals. The antioxidant system plays an important role in preventing oxidative stress and scavenging superoxide anions and hydroxyl radicals in fish [34]. In the present study, histidine deficiency significantly suppressed the intestinal antioxidant enzyme activities of T-SOD, CAT, and GPx and the intestinal level of GSH. Furthermore, the activity of antioxidant enzymes is regulated by the expression of antioxidant genes [35]. The Nrf2-signalling pathway regulates antioxidant genes [13,15]. In this study, dietary histidine deficiency significantly lowered the mRNA level of *nrf2* and increased the mRNA level of *keap1*, which further lowered the mRNA levels of the downstream genes *sod*, *cat,* and *gpx*. Thus, dietary histidine supplementation has a positive effect on these antioxidant indices and the mRNA levels of antioxidant genes, which further lowers intestinal MDA levels. Jiang et al. [13] reported that dietary histidine deficiency suppressed the antioxidant index and gene expression in the gills of grass carp. Furthermore, similar results were reported in the muscles of grass carp [15]. The results here indicated that dietary histidine deficiency increased MDA levels, which might be partially related to the decrease of antioxidant enzymes and factors in the intestine. In our previous study, dietary histidine deficiency resulted in reduced growth performance [24]. In this study, dietary histidine deficiency also had negative effects on intestinal antioxidant capacity of largemouth bass. These results might be one of the reasons why histidine deficiency could lower the growth performance of largemouth bass. Lemire et al. [36] reported that glutamate, a product of histidine catabolism, was involved in the generation of the antioxidant α-ketoglutarate, which is also considered a crucial molecule in cellular redox regulation [36,37,38,39,40]. In aquatic animals, α-ketoglutarate supplementation in the diet enhanced the antioxidant capacity in grass carp [41,42], hybrid sturgeon (*Acipenser schrenckii* ♀× *A. baerii ♂*) [43], Songpu mirror carp [44], and common carp [45]. Hence, histidine exerts its antioxidant functions through its metabolite α-ketoglutaric acid. In this study, no significant changes in T-AOC were found with different DHLs. However, only the highest DHL (1.42%) significantly increased the activity of T-AOC in juvenile blunt snout bream [46]. In this study, the DHL in the control group was 0.71%, which was much higher than that in the juvenile blunt snout bream. Therefore, it may be related to the difference in DHL in this study. Unfortunately, there are few studies on the relationship between histidine and T-AOC in aquatic animals, and the related mechanism is still unclear, which needs to be further studied. Furthermore, the recommended histidine requirement of juvenile largemouth bass was 1.26% of the diet (2.68% of dietary protein) based on the growth performance in our previous study [24]. However, in this study, the dietary histidine requirements of juvenile largemouth bass were 1.32% of the diet (2.81% dietary protein) and 1.47% of the diet (3.13% dietary protein) based on the MDA and T-SOD results, which showed that the requirement based on antioxidant capacity was slightly higher than that based on growth performance, indicating that the level of histidine in the feed might be necessary to be further increase if a better immune effect is pursued.

### 4.2. Histidine Deficiency Suppressed Intestinal Immunocompetence

Immune globulin, the interferon system, and the complement system play an indispensable role in the regulation of immune capacity [47,48,49]. In this study, dietary histidine deficiency inhibited the level of C3 compared with that after appropriate histidine supplementation, indicating that dietary histidine deficiency has a negative effect on immune regulation in largemouth bass. It has been found that the active site of C3 in all animal species contains a conserved histidine residue, which can activate the thioester bond of C3 and promote covalent binding of the activated group with the hydroxyl group on the surface of target cells, leading to the occurrence of the corresponding biochemical reaction. If this key histidine residue is substituted, complement activation cannot be completed [50]. This might be a possible reason why dietary histidine deficiency decreased the production of intestinal C3. However, dietary histidine supplementation had no significant effect on IgM, HSP70, or IFN-γ. Interestingly, only a limited number of studies have reported the regulation of immune globulin, the interferon system, and the complement system in animals. Therefore, the specific regulatory mechanism needs to be further studied.

### 4.3. Histidine Deficiency Induces Intestinal Endoplasmic-Reticulum Stress Resulting in Inflammatory Response, Apoptosis, and Necroptosis

The ER is one of the most important organelles in eukaryotic cells and is closely related to cell homeostasis, and ER homeostasis is maintained through unfolded protein reactions involving three signalling pathways, including the PEPK-signalling pathway, IRE-1-signalling pathway, and ATF6-signalling pathway [16]. In this study, dietary histidine deficiency activated the PEPK-signalling pathway and IRE1-signalling pathway with increased core gene expression of *pepk*, *grp78*, *eif2α*, *atf4*, *chopα*, *ire1*, *xbp1*, *traf2*, *ask1,* and *jnk1*, indicating that dietary histidine deficiency induced ERS. In aquatic animals, activation of the PEPK-signalling pathway and IRE1-signalling pathway resulted in ERS under heat stress in largemouth bass [32]. Furthermore, numerous studies have reported that adverse factors can induce ERS by affecting the expression of the core genes in the IRE1- and PERK-signalling pathways in blunt snout bream [17], common carp [18,19], Gibel carp [20], and grass carp [21]. However, dietary histidine deficiency had no significant effect on the mRNA level of ATF6. In contrast to this study, ERS regulation was also involved in the ATF6-signalling pathway [32,51]. Activation of the IRE1 pathway by B-cell differentiation did not lead to the upregulation of the CHOP downstream genes in the ATF6 and PERK pathways [52,53]. Therefore, it was speculated that these three pathways could be selectively activated. However, the mechanism of selection was unclear.

In this study, dietary histidine deficiency significantly upregulated the mRNA levels of proapoptotic genes, including *caspase 3*, *bax*, *caspase 8, caspase 9,* and the mRNA levels of necroptosis genes, including *mlkl* and *ripk3*. Furthermore, dietary histidine deficiency also significantly lowered the mRNA levels of the antiapoptotic gene *bcl-2*. These results showed that chronic dietary histidine deficiency could induce apoptosis and necroptosis of the intestine. Jiang et al. [13] also reported that dietary histidine deficiency induced apoptosis in the gills of grass carp by improving the mRNA levels of *caspase 3*, *caspase 8,* and *caspase 9*, which supports the results of this study. When intense or persistent ERS occurs, CHOP gene expression is activated in the three above-mentioned pathways and ERS exceeds the range of cellular self-regulation, which leads to cell apoptosis and cell death [23]. Hence, chronic dietary histidine deficiency induced persistent ERS, which further led to cell apoptosis and necroptosis in largemouth bass. Furthermore, studies have reported that IRE1 binds with TRAF2 to activate ASK1 and further activate JNK, which upregulates CHOP gene expression to regulate antiapoptotic genes and caspase-mediated apoptosis pathways [54,55]. This might be another mechanism by which dietary histidine deficiency induces apoptosis. NF-κB is an important signalling pathway in inflammatory regulation [56]. In this study, dietary histidine deficiency activated the NF-κB-signalling pathway to induce an inflammatory response, which further upregulated the mRNA levels of the proinflammatory factors *tnf-α*, *hepcidin 1*, *cox2*, *cd80*, and *cd83* and lowered the mRNA levels of the anti-inflammatory factors *tgf-β1* and *ikbα*. Furthermore, dietary histidine deficiency significantly lowered the levels of the intestinal anti-inflammatory factors IL-10 and TGF-β and upregulated the level of the intestinal proinflammatory factor TNF-α, showing a trend similar to that of the gene expression of inflammatory factors. A similar study reported that dietary histidine deficiency induced an inflammatory response in the gills of grass carp [13] and intestines of blunt snout bream [46]. Furthermore, dietary amino acid deficiency can also induce inflammatory responses, such as through methionine and lysine, in blunt snout bream [5,57]. It was reported that activation of the IRE1–TRAF2–ASK1–JNK1-signalling pathway could upregulate NF-κB and mediate the inflammatory response [23]. Dietary histidine deficiency can also activate the IRE1–TRAF2–ASK1–JNK1-signalling pathway, which might be the mechanism by which histidine regulates the NF-κB-signalling pathway. However, unfortunately, there are relatively few studies on histidine at present, and the relevant mechanisms need further study.

## 5. Conclusions

Histidine deficiency can weaken the intestinal antioxidant capacity and induce intestinal endoplasmic-reticulum stress, which further leads to an inflammatory response, apoptosis, and necroptosis and decreases the immune capacity of largemouth bass. However, histidine supplementation could alleviate these problems. Based on the results of MDA and T-SOD, the appropriate dietary histidine requirements of juvenile largemouth bass are 1.32% of the diet (2.81% dietary protein) and 1.47% of the diet (3.13% dietary protein), respectively, as determined by quadratic regression analysis.

## Figures and Tables

**Table 1:** Ingredients and proximate compositions of experimental diets (% dry matter) ^1^.

Ingredient	Diet 1 (%)	Diet 2 (%)	Diet 3 (%)	Diet 4 (%)	Diet 5 (%)	Diet 6 (%)
Fish meal ^2^	30	30	30	30	30	30
Rapeseed meal ^2^	8	8	8	8	8	8
Soybean meal ^2^	10	10	10	10	10	10
Wheat meal ^2^	16	16	16	16	16	16
Fish oil	5	5	5	5	5	5
Sleeve-fish ointment	2	2	2	2	2	2
Amino acid mixes ^3^	13.21	13.21	13.21	13.21	13.21	13.21
Choline chloride	0.1	0.1	0.1	0.1	0.1	0.1
Vitamin premix ^4^	1	1	1	1	1	1
Mineral premix ^4^	1	1	1	1	1	1
Monocalcium phosphate	2.5	2.5	2.5	2.5	2.5	2.5
Microcrystalline cellulose	3.18	3.18	3.18	3.18	3.18	3.18
Rice bran	7	7	7	7	7	7
Ethoxylquinine	0.01	0.01	0.01	0.01	0.01	0.01
Glycine	1	0.8	0.6	0.4	0.2	0
L-histidine	0	0.2	0.4	0.6	0.8	1
Compositional analysis (dry matter)						
Crude protein (%)	46.93	47.01	47.11	46.83	46.91	47.03
Crude lipid (%)	11.02	10.98	10.94	11.01	10.97	10.99
Energy (MJ/kg)	18.87	18.85	18.83	18.85	18.91	18.88
Histidine levels (%)	0.71	0.89	1.08	1.26	1.48	1.67

^1^ The experimental formulation referred to our previous study [24]; ^2^ Fish meal, rapeseed meal, soybean meal, wheat meal obtained from Wuxi Tongwei feedstuffs Co., Ltd. (Wuxi, China), crude protein 65.6%, 39.2%, 39.2%, and 13.1%, respectively; crude lipid 9.5%, 6.1%, 4.3%, and 4.0%, respectively; ^3^ Amino acid mixes: the amino acid content was balanced according to the amino acid composition of 47% protein level of the whole fish. Arginine, 0.87%; isoleucine, 0.74%; leucine, 1.21%; lysine, 1.40%; methionine, 0.37%; phenylalanine, 0.61%; threonine, 0.74%; valine, 0.74%; tryptophan, 0.14%; aspartic acid, 1.00%; serine, 0.71%; glycine, 1.16%; alanine, 0.52%; glutamic acid, 2.02%; proline, 0.98%. All amino acids obtained from Feeder Co., Ltd. (Shanghai, China); ^4^ Vitamins premix and mineral mix were purchased by HANOVE Biotechnology Co., Ltd. (Wuxi, China).

**Table 2:** Primer sequences for RT-qPCR.

Gene Name	Forward Sequence	Reverse Sequence	Amplification Efficiency (%)	Source
*nrf2*	CCACACGTGACTCTGATTTCTC	TCCTCCATGACCTTGAAGCAT	102.5	[27]
*keap1*	GCACCTAACCGTGGAACTCAA	CCAGTTTTAGCCAGTCATTGTTCC	99.8	
*cat*	TGGTGTTCACGGATGAGATGG	GGAGAAGCGGACAGCAATAGG	98.6	
*sod*	CCACCAGAGGTCTCACAGCA	CCACTGAACCGAAGAAGGACT	101.2	
*gpx*	CCCTGCAATCAGTTTGGACA	TTGGTTCAAAGCCATTCCCT	102.5	[28]
*nf-κb*	CCACTCAGGTGTTGGAGCTT	TCCAGAGCACGACACACTTC	100.8	XP_027136364.1
*tnf-α*	CTTCGTCTACAGCCAGGCATCG	TTTGGCACACCGACCTCACC	99.9	[29]
*il-8*	CGTTGAACAGACTGGGAGAGATG	AGTGGGATGGCTTCATTATCTTGT	103.6	
*il-1β*	CGTGACTGACAGCAAAAAGAGG	GATGCCCAGAGCCACAGTTC	103.4	
*il-10*	CGGCACAGAAATCCCAGAGC	CAGCAGGCTCACAAAATAAACATCT	101.1	
*tgf-β1*	GCTCAAAGAGAGCGAGGATG	TCCTCTACCATTCGCAATCC	98.5	
*hepcidin1*	CATTCACCGGGGTGCAA	CCTGATGTGATTTGGCATCATC	99.4	[28]
*cox2*	CACTGGGTCGTGTCACTTT	TGATTCTCCTCCTTGCTGT	101.3	[30]
*cd80*	TCTTCATCGTGGTAATAATAGG	TGTGGTGTCTTCAGGGTCT	98.9	
*cd83*	CACTGTTGTGCCTTGCTG	GGAGCCTCTTTGACCTTGT	99.8	
*ikbα*	CCCCAACTACAGTGGACAAA	AAGGTCAAGGAGGCAACG	103.1	
*caspase 3*	GAGGCGATGGACAAGAGTCA	CACAGACGAATGAAGCGTGG	99.8	XM_038713063.1
*bcl-xl*	CATCCTCCTTGGCTCTGG	GGGTCTGTTTGCCTTTGG	103.5	[31]
*caspase 8*	GAGACAGACAGCAGACAACCA	TTCCATTTCAGCAAACACATC	101.8	[28]
*caspase 9*	CTGGAATGCCTTCAGGAGACGGG	GGGAGGGGCAAGACAACAGGGTG	99.7	
*bcl-2*	CGCCATCCACAGAGTCCT	CCGGAACAGTTCGTCTATCACC	101.1	[30]
*bax*	ACTTTGGATTACCTGCGGGA	TGCCAGAAATCAGGAGCAGA	101.9	
*mlkl*	CCCAAGCCTCAGTTCCTC	TTTCTTCGGTCTGGTGCA	102.1	
*tnrf1a*	GCATACCCAGAATGTGAGA	CATAACCGCCACGACTAA	99.3	
*ripk3*	GTTTAGGGCAGGAGGTGA	TTCTGAGTTTCCCAATGTTT	99.7	
*eif2α*	CCTCGTTTGTCCGTCTGTATC	GCTGACTCTGTCGGCCTTG	101.2	[28]
*traf2*	CTGCCAAACCTTAATCCTT	ACAGACTTACAGCCCACTTC	99.5	
*xbp1*	ACACCCTCGACACGAAAGA	AGAATGCCCAGTAGCAAATC	98.9	[30]
*grp78*	TTGCCGATGACGACGAAA	CAATCAGACGCTCACCCT	102.1	
*chopα*	GATGAGCAGCCTAAGCCACG	AACAGGTCAGCCAAGAAGTCG	101.5	
*perk*	CCACCGCAGAGCAGATGTAA	TGCTGGAGTCATCCTACCGA	102.7	[32]
*ask1*	CAACTACGCCTTCATCCCGT	GGTCCCAACAGCATCTCGAA	99.7	
*ire1*	CTGCCAGATCCGCATACACT	GGTGTCCACTCTTGAAGGCA	98.5	
*atf6*	GACGCCCCGCATAAGAGTAA	GCAGACTTGAGGAGAGCTGG	101.6	
*jnk1*	TGCACTACCTGAGCCACTTG	TGTGCTTCCTGGCTGATGTT	100.3	XM_038735152.1
*atf4*	GCGGACATTTGTGTTGCACT	CTGTCCTGCCAGGTGATGAA	99.2	XM_038712790.1
*gapdh*	ACTGTCACTCCTCCATCTT	CACGGTTGCTGTATCCAA		AZA04761.1

## Data Availability

All datasets generated for this study are available.
